# Glioblastoma Models Reveal the Connection between Adult Glial Progenitors and the Proneural Phenotype

**DOI:** 10.1371/journal.pone.0020041

**Published:** 2011-05-23

**Authors:** Liang Lei, Adam M. Sonabend, Paolo Guarnieri, Craig Soderquist, Thomas Ludwig, Steven Rosenfeld, Jeffrey N. Bruce, Peter Canoll

**Affiliations:** 1 Department of Pathology and Cell Biology, Columbia University, New York, New York, United States of America; 2 Department of Neurological Surgery, Columbia University, New York, New York, United States of America; 3 Biomedical Informatics Shared Resources, Bioinformatics Division, Columbia University, New York, New York, United States of America; 4 Department of Neurology, Columbia University, New York, New York, United States of America; 5 Herbert Irving Comprehensive Cancer Center, Columbia University, New York, New York, United States of America; The University of Chicago, United States of America

## Abstract

**Background:**

Tumor heterogeneity is a major obstacle for finding effective treatment of Glioblastoma (GBM). Based on global expression analysis, GBM can be classified into distinct subtypes: Proneural, Neural, Classical and Mesenchymal. The signatures of these different tumor subtypes may reflect the phenotypes of cells giving rise to them. However, the experimental evidence connecting any specific subtype of GBM to particular cells of origin is lacking. In addition, it is unclear how different genetic alterations interact with cells of origin in determining tumor heterogeneity. This issue cannot be addressed by studying end-stage human tumors.

**Methodology/Principal Findings:**

To address this issue, we used retroviruses to deliver transforming genetic lesions to glial progenitors in adult mouse brain. We compared the resulting tumors to human GBM. We found that different initiating genetic lesions gave rise to tumors with different growth rates. However all mouse tumors closely resembled the human Proneural GBM. Comparative analysis of these mouse tumors allowed us to identify a set of genes whose expression in humans with Proneural GBM correlates with survival.

**Conclusions/Significance:**

This study offers insights into the relationship between adult glial progenitors and Proneural GBM, and allows us to identify molecular alterations that lead to more aggressive tumor growth. In addition, we present a new preclinical model that can be used to test treatments directed at a specific type of GBM in future studies.

## Introduction

Glioblastoma (GBM) is the most common and most malignant type of primary brain tumor. It represents one of the deadliest human cancers, with average survival at diagnosis slightly over one year. GBM is remarkably heterogeneous, and may actually represent several distinct entities with different cells of origin, different genetic lesions and different clinical behaviors [1], [2]. Numerous studies have characterized the expression profiles and genetic alterations found in GBM [3]–[9]. A recent comprehensive analysis of The Cancer Genome Atlas (TCGA) dataset has identified four distinct subtypes of GBM; Proneural, Neural, Classical and Mesenchymal [10]. Interestingly, each of these subtypes show an enrichment of gene expression signatures from distinct neural lineages, implying that the expression patterns of the different subtypes may reflect the phenotype of their specific cells of origin [10].

A number of studies have used animal models to explore the process of gliomagenesis [11]–[27]. Many of these models induced tumor formation by introducing genetic lesions into the embryonic or neonatal brain. These various models have given rise to different tumor types, including oligodendroglioma, astrocytoma and GBM. It is not clear how the genetic alterations and/or the cells of origin to which these alterations were introduced influence tumor phenotype in these models. Similarly, it is unclear how age affects the phenotypic and tumorigenic potential of progenitor cells. This is relevant to the human situation, since the majority of GBM occurs in adults, which implies that the cells giving rise to these tumors reside in the adult brain.

There are several different populations of cells in the adult brain that may have the capacity to form brain tumors, including neural stem cells in the subventricular zone (SVZ) [18], [25], [26] and more differentiated glial progenitors in the subcortical white matter [17], [28]. Furthermore, experiments using the RCAS/tv-a system have shown that progenitors with the capacity to form tumors are not restricted to the SVZ, but are distributed throughout the adult brain [29]. Among the adult glial progenitor populations, the best characterized are the oligodendrocyte progenitor cells (OPCs) that express PDGFRα, NG2 and Olig2 [28], [30]–[32]. OPCs are widely distributed, both in the white matter and cortex, and comprise the largest population of cycling cells in the adult brain [33]–[37]. Thus OPCs represent an abundant reservoir of potentially transformable cells.

Different progenitor populations may utilize different mechanisms to regulate proliferation, differentiation and survival. Therefore, the genetic alterations that are required to transform them may also differ. In support of this idea, studies looking at brain tumors that commonly occur in children (ependymomas and medulloblastomas) suggested that certain subtypes of these brain tumors arise from discrete populations of neural progenitor cells in the embryonic brain. Furthermore, these different progenitor cells are susceptible to the genetic alterations seen in the particular tumor subtype to which they give rise [38], [39]. Specific genetic lesions are also observed in different subtypes of GBM. Mutations and loss of heterozygosity of p53 and amplification of PDGFRα are most frequently seen in the Proneural subtype, while loss of Pten is observed throughout all subtypes [8]–[10], [40]. However, much remains to be learned about how the cells of origin and genetic alterations interact to determine GBM phenotype.

In this study, we have approached this question by developing mouse models for GBM, which combine retroviral delivery of oncogenes with conditional deletions of tumor suppressor genes. This approach has the unique advantages of allowing us to control the time and location of tumor initiation. Using retroviruses expressing PDGF and Cre recombinase, we have stereotactically delivered transforming genetic alterations to a discrete population of progenitors in the subcortical white matter of adult mice that harbor floxed tumor suppressor genes. Fate mapping showed that the retrovirus selectively infects a local population of glial progenitor cells that predominantly give rise to cells of the oligodendrocyte lineage. However, we found that the combination of PDGF expression with genetic deletion of Pten and p53 induces these cells to form GBM-like brain tumors with 100% penetrance. Our results revealed that the mouse tumors closely resemble the Proneural subtype of human GBM. Furthermore, both the mouse tumors and Proneural GBM are highly enriched in genes expressed by OPCs, providing further evidence for the close relationship between OPCs and the Proneural GBM. Finally, using a set of genes that are differentially expressed between mouse models with different genetic deletions (Pten^f/f^ vs. Pten^f/f^; p53^f/f^), we are able to separate human Proneural GBM into groups with different molecular and clinical features. Together, these findings indicate that glial progenitors that reside in the adult white matter can form brain tumors that recapitulate both the genomic and phenotypic profile seen in a specific subtype of human GBM.

## Results

### PIC retrovirus induces GBM-like tumors in mice with conditional Pten and p53 alleles

**Figure 1:**
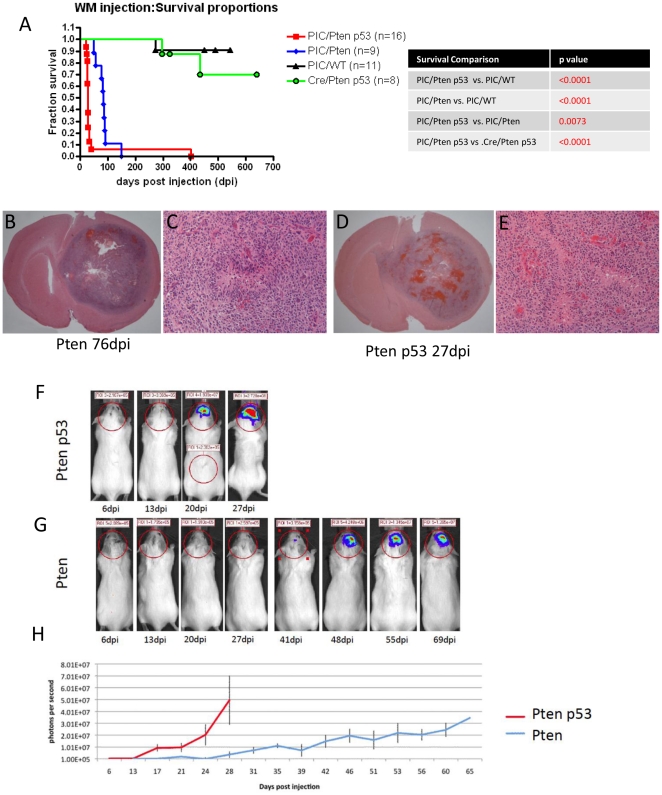
PDGF-IRES-Cre retrovirus induced tumor formation in mice with floxed Pten and p53. (A) Kaplan-Meier curves comparing the survival of mice from our 4 experimental groups: All PIC injected Pten^f/f^ mice and Pten^f/f^; p53^f/f^ mice developed brain tumors with median survivals of 85 dpi and 27 dpi, respectively. Three mice from the control groups died from unrelated causes during the study. The remaining control mice were sacrificed and analyzed at the end of the study, none showed evidence of tumor. The survival differences between all groups were highly significant. The p values for all comparisons are shown in the right panel. (B–E) Low and high powered micrographs showing H & E stains of end stage tumors in Pten^f/f^ mice (B and C) and Pten^f/f^; p53^f/f^ mice (D and E). Both tumors show histological features of GBM, including areas of pseudopalisading necrosis (N). (F–G) Bioluminescence imaging of tumor growth in Pten^f/f^; p53^f/f^ (F) and Pten^f/f^ (G) mice. (H) Plot showing the different growth rates of tumors in Pten^f/f^ (blue line) and Pten^f/f^; p53^f/f^ (red line) mice. Each line shows the changes in the mean value of the luciferase signal of 11 mice per group. Bars show the S.E.M at each time point.

We injected VSVG-pseudotyped PDGF-IRES-Cre (PIC) retrovirus, which expresses PDGF and Cre in one transcript, into rostral subcortical white matter (WM) of transgenic mice that carry floxed Pten (Pten^f/f^) or floxed Pten and p53 (Pten^f/f^; p53^f/f^). Brain tumors with the histological features of human GBM formed with 100% penetrance in both genotypes (Figure 1A–E). However, the rate of tumor formation differed significantly between these two genotypes: mice with floxed Pten and p53 (Pten^f/f^; p53^f/f^) had a median survival of 27 days post injection (dpi) compared to 85 dpi for mice with floxed Pten (Pten^f/f^). These mice also harbored a stop-floxed luciferase reporter, as a consequence, only retrovirus infected cells and their progeny expressed luciferase, allowing us to monitor tumor growth by bioluminescence imaging. Consistent with the results of our survival study, the two models showed marked differences in tumor growth dynamics, with the tumors in Pten^f/f^; p53^f/f^ mice developing earlier and growing faster than those in Pten^f/f^ mice (Figure 1F–H). As controls, we injected PIC retrovirus into stop-flox luciferase mice with wild type Pten and p53 and Cre-only retrovirus (no PDGF) into Pten^f/f^; p53^f/f^ mice. No luciferase signal was ever detected in the control mice (data not shown), and none of the control mice ever developed tumor related morbidity (Figure 1A). These results demonstrate that, when given alone, neither PDGF expression nor deletion of Pten and p53 is sufficient to induce tumor formation. However, the combination of PDGF stimulation and deletion of Pten and p53 cooperate to induce tumor formation with remarkable robustness and consistency.

**Figure 2:**
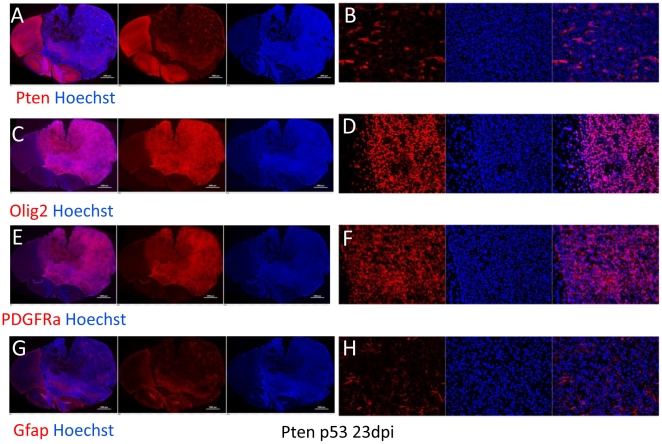
Immunophenotype of end stage tumors is consistent with OPC identity. Each triptych shows the immunostaining (red), Hoechst nuclear stain (blue) and combined for low power (left) and high power (right) micrographs of ends-stage tumors in Pten^f/f^; p53^f/f^ mice. (A and B) Most cells in the tumor lacked Pten staining. (C–F)The vast majority of tumor cells express olig2 (C and D) and PDGFRα (E and F). (G and H) GFAP+ astrocytes were scattered throughout the tumor, but represented a minority of cells.

Tumors from Pten^f/f^; p53^f/f^ mice are predominantly composed of Pten negative cells, and express high levels of Olig2 and PDGFRα, markers normally expressed by OPCs (Figure 2A–F). Many GFAP+ cells are scattered throughout the tumor mass (Figure 2G and H), and represent entrapped astrocytes (see below). Similar results were seen with tumors from Pten^f/f^ mice (data not shown).

Primary tumor preparations were made from tumors in Pten^f/f^ and Pten^f/f^; p53^f/f^ mice. These cells were serially transplanted intracranially in NOD/SCID mice for up to four generations. We found that the transplanted cells continue to give rise to tumors that have the histological features of GBM, with an immunophenotype similar to that of the primary tumor (Figure S1). Thus, the retrovirus-infected progenitor cells are fully transformed and possess tumorigenic potential with extensive capacity to self-renew. Furthermore, these cells can be propagated in cultures using growth conditions that have been optimized for OPCs [17], [41]. Immunocytochemical analysis confirmed that these cells continue to express many OPC markers, including Olig2, PDGFRα, Nkx2.2, NG2 and Sox10 (Figure S2) as well as markers seen in glial progenitors and other progenitor populations, such as Sox2, Nestin and Mash1 (data not shown).

### Early stage tumors are composed of proliferating OPCs

**Figure 3:**
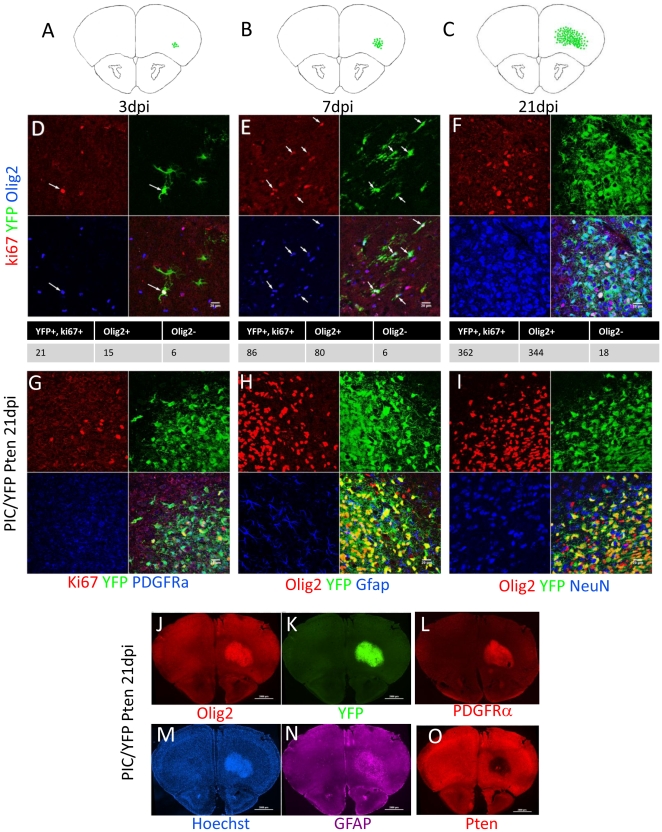
Phenotypes of early tumor lesions in YFP; Pten^f/f^ mice indicated OPC identity. (A–C) Schematic views show the distribution of YFP+ cells (green dots) at 3 dpi (A), 7 dpi (B) and 21 dpi (C). (D–F) Triple immunofluorescence shows YFP (green) ki67 (red) and Olig2 (blue) at 3 dpi (D), 7 dpi (E) and 21 dpi (F). The arrows in D and E mark triple positive cells. The tables below each panel show the counts of ki67+/YFP+ cells at each time point (total, Olig2+, and Olig2−). (G–I) Triple immunofluorescence shows the expression of different markers in tumor cells at 21 dpi: (G) shows ki67 (red), YFP+ (green) and PDGFRα+ (blue). (H) shows Olig2 (red), YFP (green) and GFAP (blue). (I) shows Olig2 (red), YFP (green) and NeuN (blue). (J–O) Low power montage of 21 dpi tumor shows Olig2 (J), YFP (K), PDGFRα (L), Hoechst (M), GFAP (N) and Pten (O).

To characterize the phenotype of cells giving rise to these tumors, we injected PIC retrovirus into mice with floxed Pten and stop-floxed YFP. In these mice, the retrovirus infected cells showed both Cre mediated deletion of Pten and Cre induced expression of YFP. The YFP reporter enabled us to identify cells infected by retrovirus and monitor tumor growth from very early stages. At 3 dpi, a small collection of YFP+ cells was seen at the injection site (Figure 3A). The YFP+ population was progressively larger at later time points (7 dpi and 21 dpi) with many YFP+/Pten- cells infiltrating the surrounding brain tissue (Figure 3B and C). At 3 dpi, more than 70% of the proliferating, retrovirus-infected cells (YFP and ki67 double positive cells) expressed Olig2 (Figure 3D). This fraction increased over time, reaching over 90% by 7 dpi and over 95% by 21 dpi (Figure 3E and F). Interestingly, at early time points we observed some Ki67+ cells that did not express YFP. These cells likely represent uninfected glial progenitors that are being induced to proliferate via paracrine growth factor stimulation, as previously described [17], [22], [42]. However, the YFP- cells at 21 dpi accounted for only ∼6% of total number of proliferating cells (data not shown). This indicates that the retrovirus infected cells, with Pten deleted, have a significant selective advantage, and quickly become the predominant population within the tumor.

We found that the growing lesion is always centered at the injection site, demonstrating that the tumor arises from locally infected cells. By 21 dpi, there is already a well-defined hypercellular lesion (Figure 3J–O). These tumors are predominantly composed of Olig2+/PDGFRα+/YFP+/Pten- cells (Figure 3G–O). Furthermore, the majority of Ki67+ cycling cells are also positive for Olig2, PDGFRα, and YFP+ (Figure 3F and G), demonstrating that the tumors were arising from retrovirus-infected cells that resemble proliferating OPCs. In addition, we observed many GFAP+ reactive astrocytes scattered throughout the tumor. However, none of these cells are YFP+, ki67+ or Phospho-histone H3 (PHH3)+ (Figure 3H, K, N and data not shown), suggesting that the GFAP+ cells are not derived from virally infected cells and do not represent a neoplastic population. We also observed that as YFP+ cells infiltrate the surrounding brain tissue they become intermingled with NeuN+ neurons. However, none of the YFP+ cells express the neuronal marker NeuN (Figure 3I).

To characterize the fate of the retrovirus-infected cells when Pten was not deleted, we injected PIC retrovirus into subcortical white matter of mice with wild type Pten and stop-floxed YFP (YFP mice). We observed that at all time points (3, 17, 94 and 143 dpi), YFP+ cells remain clustered around the injection site. Immunohistochemical staining for Olig2, PDGFRα and GFAP shows that the YFP+ populations initially contains a mixture of astrocytes and OPCs at 3 dpi (Figure S3A). However, by 17 dpi, more than 70% of YFP+ cells express Olig2 (Figure S3B). As early as 3 dpi, OPCs account for the majority of Ki67+ cycling cells (Figure S3C and D). Over time, the Olig2+/PDGFRα+/YFP+ population slowly expands around the injection site and becomes the predominant population (Figure S3E and F). We never observed YFP+ cells in the subventricular zone (SVZ), rostral migratory stream (RMS) or olfactory bulb (OB) (Figure S3F and data not shown), confirming that retrovirus injections into subcortical white matter selectively infect a local population of glial progenitors. In contrast, with PIC retrovirus injection into the SVZ of the lateral ventricle in YFP mice, many YFP+ cells are seen in the SVZ, RMS and OB, where some of them eventually differentiate into neurons (Figure S4). Together, these results demonstrate that injecting the PIC retrovirus into the subcortical white matter selectively infects local glial progenitors, and it is this population that gives rise to brain tumors.

### The retrovirus driven mouse tumors resemble human Proneural GBM

**Figure 4:**
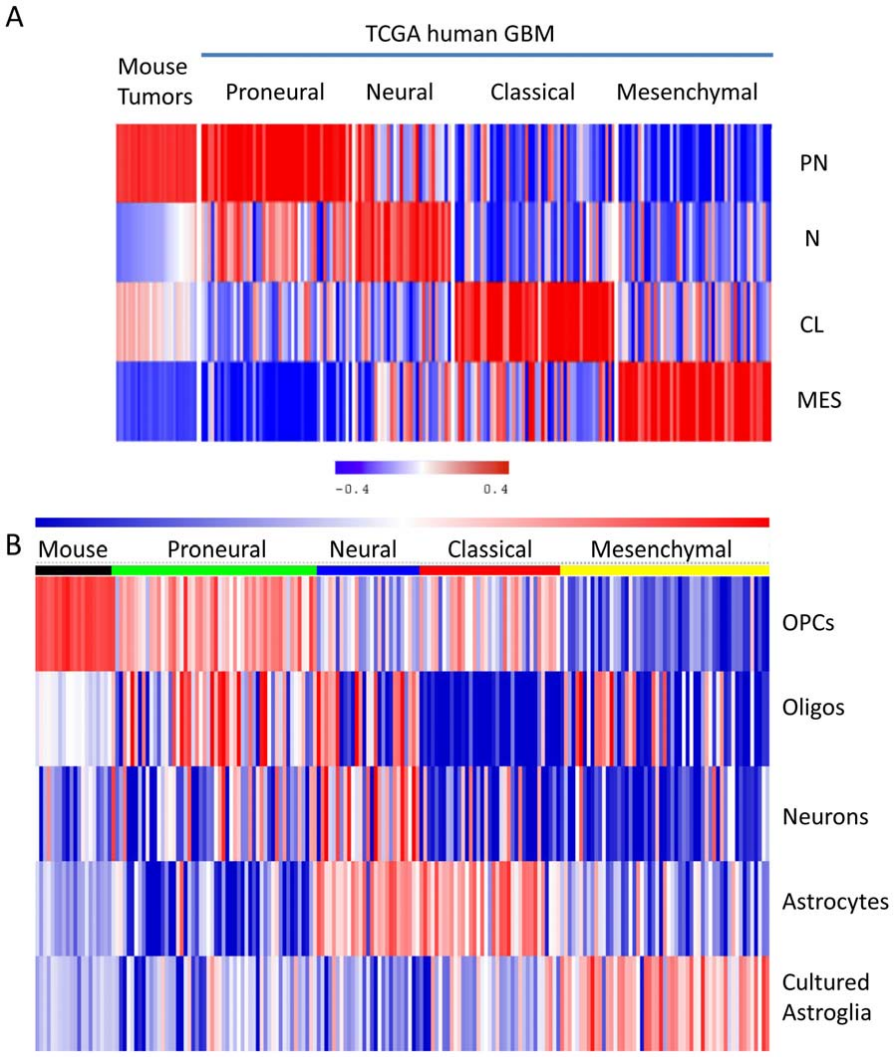
PDGF driven mouse tumors most resembled human Proneural GBM and express signatures of OPCs. (A) Heat map of the Pearson correlation based classification of mouse and human GBM samples. Red to blue color scale shows the range from the highest positive to highest negative correlation. The correlation was computed for both mouse samples and TCGA human GBM. A sample is assigned the class with the highest correlation coefficients (see Methods for details). (B) Mouse tumors and human Proneural patients were both enriched with OPC genes. Sample wise: mouse tumors are labeled as black, Proneural as green, Neural as blue, Classical as red and Mesenchymal as yellow. Red to blue color scale shows the range from the highest to lowest enrichment score. These genes lists are provided in Table S1.

We compared the relationship of the mouse tumors and human GBM at the molecular scale. Gene expression profiling was performed on 20 tumors from mice (12 Pten^f/f^ and 8 Pten^f/f^; p53^f/f^), which were compared to 218 human GBM from TCGA. Of 840 genes that were used to classify human GBM from TCGA into 4 subtypes (Proneural, Neural, Classical and Mesenchymal) [10], 723 orthologs could be mapped to the mouse arrays (see Methods and Table S1). We adapted the centroid-based classifier in order to use these orthologs genes [10]. This new classifier was re-trained and re-tested on the original TCGA dataset, with the loss in classification power was less than 10% (Table S1). The new classifier also retained the capability to sort the same TCGA dataset into four subtypes (Figure 4A and Figure S5A). Remarkably, all mouse tumors were classified as Proneural using this modified classifier (Figure 4A, Table S1). Using these 723 genes, hierarchical clustering showed that mouse tumors preferentially clustered with the Proneural GBM (Figure S5A), providing an additional support that these mouse tumors resemble human Proneural GBM.

### Both Mouse tumors and human Proneural GBM are enriched in OPC genes

We then interrogated a murine dataset that was generated from neural lineages isolated from mouse brain [43]. From this study we compiled a list of 369 genes that were derived from 5 gene lists as published [43], which represented signatures of five cell types: OPCs, oligodendrocytes, neurons, astrocytes and cultured astroglia (see Methods and Table S1). We conducted a Gene Set Enrichment Analysis (GSEA) using this gene list on the mouse tumors and human tumors from the TCGA dataset. Both mouse tumors and human Proneural GBM were enriched with the gene signature of OPCs (Figure 4B). Some, but not all, Proneural patients were also enriched with the gene signature of oligodendroctytes. Importantly, neither mouse tumors nor human Proneural GBM showed enrichment in genes specific for the other cell types (neuronal, astrocyte or cultured astrocyte). In contrast, each of the other subtypes of human GBM showed marked enrichment for genes expressed in one of the other cell types (Figure 4B). In addition, when sorted with this list, all mouse tumors in our dataset clustered closely with the OPC samples in the Cahoy et al. dataset (Figure S5B), providing additional indication that mouse tumors are enriched in OPC genes. As an internal control, three normal brain samples in our dataset clustered with the forebrain sample, and together as a group, clustered with neurons from the Cahoy et al. dataset (Figure S5B).

### Differential gene expression of mouse tumors revealed heterogeneity within human Proneural patients

We found that the mouse tumors from both models (Pten^f/f^ vs. Pten^f/f^; p53^f/f^) are remarkably homogeneous with respect to the expression of Proneural classifier and OPC genes (Figure 4A, B and Figure S5A, B). However, survival and imaging studies demonstrate that two models also have very different growth behaviors, with the combined deletion of Pten and p53 giving rise to more aggressive tumors (Figure 1A, F–H). To define the differences between these two models, we performed differential gene expression (DGE) analysis (see Methods) by comparing the Pten^f/f^ vs. Pten^f/f^; p53^f/f^ tumors. This analysis identified 533 differentially expressed genes; 224 genes are expressed at higher levels in Pten^f/f^; p53^f/f^ tumors compared to Pten^f/f^ tumors (we refer to this gene list as “up in Pten+p53”), and 309 genes are expressed higher in Pten^f/f^ tumors compared to Pten^f/f^; p53^f/f^ tumors (we refer to this gene list as “up in Pten”). 137 of the 224 genes in the “up in Pten+p53” list and 162 of 309 genes in the “up in Pten” list could be mapped to human microarray platform (Table S1).

**Figure 5:**
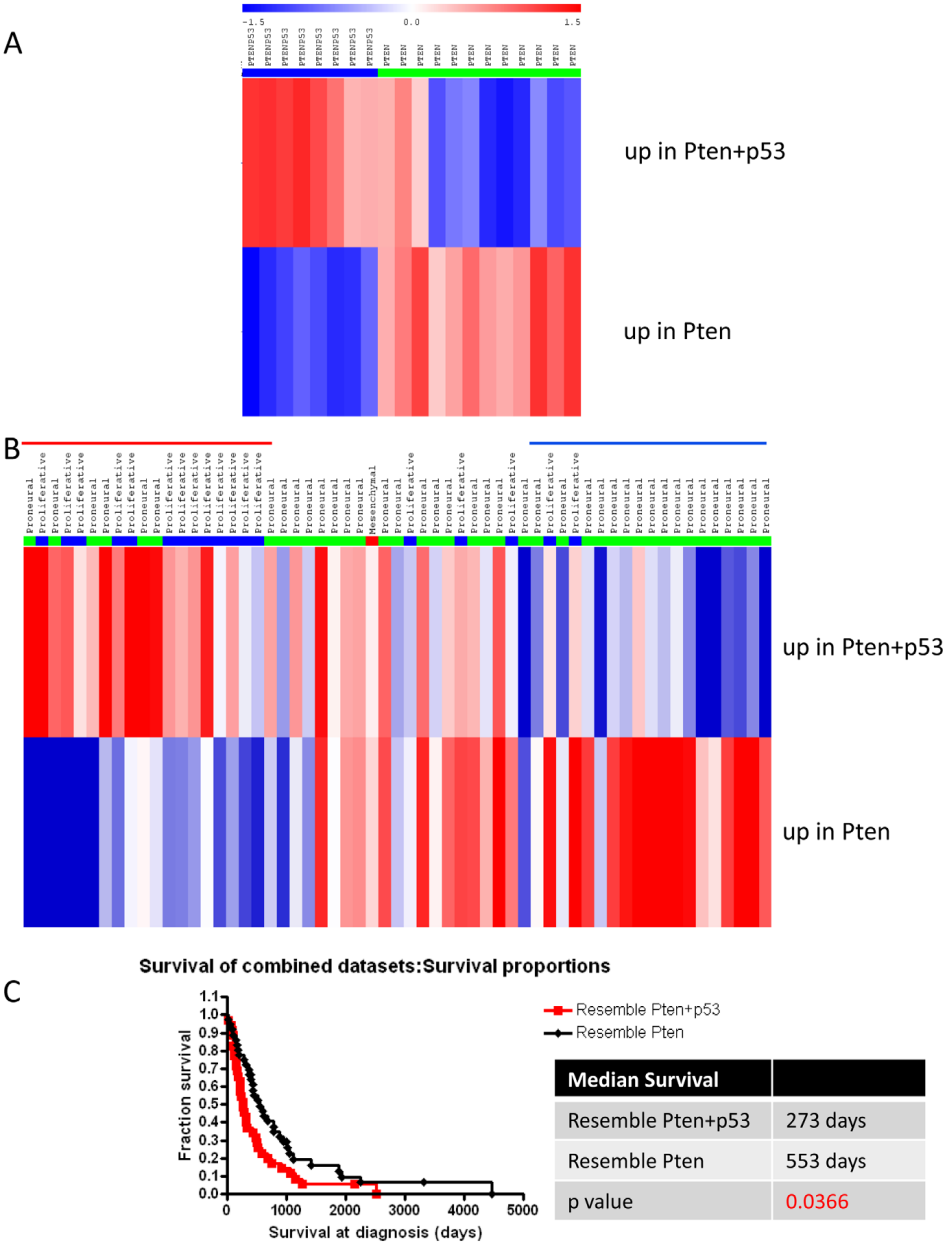
DGE lists reveal heterogeneity in human Proneural GBM. (A) GSEA of mouse tumors with DGE lists of Pten^f/f^ vs. Pten^f/f^; p53^f/f^ mouse tumors. Sample wise: Pten^f/f^; p53^f/f^ tumors were labeled as blue and Pten^f/f^ tumors as green. Red to blue color scale shows the range from the highest to lowest enrichment score. (B) GSEA ranking of TCGA Proneural patients with DGE lists of Pten^f/f^ vs. Pten^f/f^; p53^f/f^ mouse tumors. Horizontal red line on top of panel labels 1/3 of Proneural patients that most resemble Pten^f/f^; p53^f/f^ tumors, while blue line labels 1/3 of patients that most resemble Pten^f/f^ tumors. Similar patients from four datasets are pooled together to compare survival as in (C). Sample wise: Proliferative is labeled as blue, Mesenchymal as red and the rest of Proneural as green (See discussion). (C) Kaplan-Meier survival curve comparison of patients that resemble Pten^f/f^; p53^f/f^ mouse tumor vs. those that resemble Pten^f/f^ mouse tumor.

We conducted GSEA to illustrate the enrichment patterns of these two gene lists in relationship to the two mouse models. An enrichment score (ES) for each gene list was calculated for every mouse tumor. The tumors were then rank-ordered based on the difference between the ES of two gene lists. As expected, this procedure sorted the mouse tumors according to genotype, with Pten^f/f^ and Pten^f/f^; p53^f/f^ tumors showing an inverse relationship with respect to the ES for two lists of genes, and therefore falling on opposite ends of the GSEA ranking (Figure 5A). We next used the same procedure to sort the 59 TCGA human Proneural GBM into those that more closely resemble one or the other mouse models. We observed an inverse correlation in those patients at either ends of GSEA ranking: those with higher ES on the “up in Pten+p53” list tended to have lower ES on the “up in Pten” list, and vice versa (Figure 5B).

### The heterogeneity of human Proneural patients revealed by mouse DGE lists correlated with survival differences

We extended the GSEA analysis to three additional datasets [7], [44], [45]. Out of the combined 176 patients, 48 were classified as Proneural GBM (Verhaak RG, Hoadley KA and Hayes DN, personal communications). Together with 218 TCGA patients, these 394 (176+218) GBM, of which 107 (48+59) were assigned as Proneural, is comprehensive and include every GBM with the subtype known to us. Using the difference in Enrichment Score (ES), we sorted Proneural patients from each of the datasets (Table S1). One third of total patients were selected from either end of the GSEA ranking for each of the 4 datasets, because their tumors displayed signatures closest to those of Pten^f/f^ or Pten^f/f^; p53^f/f^ mouse tumors. The survival data from all these patients were pooled together. Strikingly, the patients with tumors that resembled Pten^f/f^; p53^f/f^ mouse tumors lived significantly shorter than patient with tumors that more closely resembled Pten^f/f^ tumors: median survival of 273 days vs. 553 days with p = 0.0366 (Figure 5B and C, Table S1). Thus, comparison with the mouse models allows us to separate Proneural patients into groups with different clinical outcomes.

### The DGE lists contain many genes involved in p53 signaling

The defining experimental difference between our two mouse tumor models is that one includes p53 deletion as an initiating genetic alteration, whereas the other does not.

**Figure 6:**
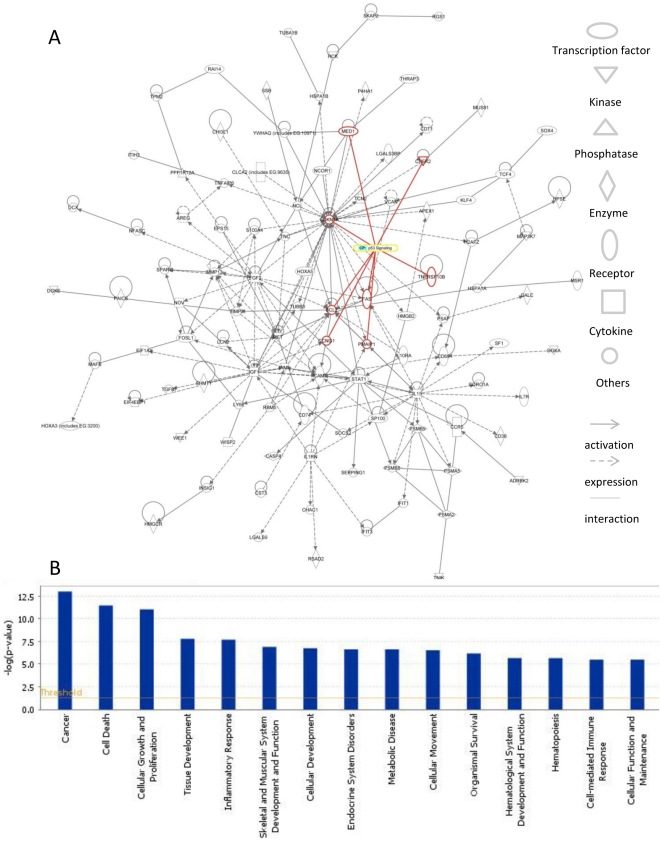
Ontology analysis reveals many genes on DGE lists are related to p53 signaling. (A) 104 members of DGE list were connected in Ingenuity pathway analysis. Out of these, 8 members of core p53 signaling pathway are highlighted as red. The shapes on the left indicate the functions and relationships of the genes. (B) The biological processes and disease pathways are significantly enriched with genes on the DGE list. The yellow line indicates the threshold to significance.

Therefore, we reasoned that Gene Ontology analysis on the combined DGE list would identify genes whose expression changes when p53 is deleted. Out of 299 genes (137+162), 8 of them are members of core p53 signaling pathway, as defined by Ingenuity Pathways Knowledge Base (see Methods). Through them, another 96 genes are connected to p53 signaling pathway (Figure 6A, Table S1). Thus, the DGE list is highly enriched for genes that are functionally related to p53. As a master transcriptional regulator, p53 can either activate or repress target gene expression [46]. Several genes on the DGE list, including Ccng1, Cdkn1a, Fas and Tnfrsf10, which contain binding sites for p53 in their promoters and whose expression is activated by p53, are expressed at higher levels in Pten^f/f^ tumors vs. Pten^f/f^; p53^f/f^ tumors. Conversely, several genes that are repressed by p53, such as Bcl2, Fgf2 and Wee1, are expressed at lower levels in Pten^f/f^ tumors. Members of the combined DGE list are also enriched in a variety of biological functions and diseases, the top of which includes cancer, cell death and growth (Figure 6B). Not surprisingly, many of these processes are tightly regulated by p53 [47], [48]. Interestingly, 195 genes on the DGE list were not identified as being related to p53 by the Ingenuity-based analysis, suggesting that such a relationship has not previously been established. We propose that these 195 genes are ideal candidates for extending new connections of p53 in relation to gliomagenesis. Together, our approach provides a powerful tool to identify genes whose altered expression leads to tumor aggressiveness.

## Discussion

### Glial progenitors in the adult subcortical white matter can give rise to brain tumors

The question of which cells can give rise to GBM, and where in the adult brain such cells reside, has long been an area of intense research and controversy [1], [2], [49], [50]. Many studies have provided evidence that neural stem cells in the SVZ have the capacity to form brain tumors [18]–[20], [25], [26], [51], [52]. However, radiographic data from patients suggests that the origins of GBM are not restricted to the SVZ and that many tumors arise in the subcortical white matter [53]. Furthermore, evidence suggested that GBM arising outside the SVZ may have distinct growth characteristics and clinical outcomes [54], [55]. Therefore, characterizing the tumorigenic potential of cells that reside outside the SVZ is of both scientific and clinical interest.

In this study, small volumes of retrovirus (0.4 µl) were injected into the rostral subcortical white matter (WM), a region that is anatomically distant from SVZ and enriched in glial progenitor cells. Retroviruses only infect dividing cells [56], and therefore, can be used to selectively target progenitor cells in the brain [57]–[59]. Using a stop-floxed YFP reporter, we were able to identify and fate map the cells that had been infected by PDGF-IRES-Cre expressing retroviruses. The distinct advantages of this approach allow us not only to control the timing and location of tumor initiation, but also to genetically label these cells and monitor tumor growth from very early stages. By 3 dpi a small collection of YFP+ cells are seen clustered around the injection site, and we found that this local population of white matter progenitor cells gives rise to the subsequent tumors.

Fate mapping showed that none of the cells infected by retrovirus injected into the subcortical white matter give rise to neurons. Rather, they exclusively give rise to cells of glial lineage, predominantly cells of the oligodendrocyte lineage. We also observed infection of some local GFAP-expressing astrocytes. We cannot completely rule out the possibility that some of these retrovirus-infected astrocytes were induced to stop expressing GFAP and adopt an OPC phenotype in response to aberrant PDGF stimulation. However, OPC-like cells were the predominant proliferating population at all time points of tumor development. Thus, our results indicate that these tumors are either arising directly from OPCs or from some closely related white matter progenitor cell that quickly acquires, and retains, an OPC-like phenotype.

### The Proneural phenotype reflects a close relationship to OPCs

GBM can be divided into subtypes on the basis of global gene expression [7], [10]. Using the same approach as published [10], we showed that the mouse tumors are most closely related to the Proneural subtype. Notably, the set of 210 classifier genes used to define the Proneural subtype contains many OPC genes, including Olig2, Nkx2.2, ErbB3, Sox4 and others (Table S1). In fact, 94 of the 210 (>45%) Proneural classifier genes are enriched in OPCs [10], [43] (Table S1). It was previously shown that Proneural GBM express genes that are associated with the oligodendrocyte lineage [10]. To further explore this issue, we expanded the analysis to compare the expression of genes that are enriched in OPCs to that of other cell types isolated from the mouse brain [43]. We showed that the mouse tumors and human Proneural GBM were both consistently enriched in OPC genes, but not in the signature genes of other cell types (oligodendrocytes, neurons, astrocytes or cultured astroglia). These results provide further evidence for the close relationship between Proneural GBM and OPCs.

### PDGF signaling is a functional link between OPCs and Proneural GBM

Certain common genetic alterations tend to occur in specific GBM subtypes [10]. This raises the possibility that these genetic alterations are particularly effective at transforming the cell type that gives rise to the specific GBM subtype. Amplification of the PDGFRα receptor is most commonly seen in Proneural GBM [2], [10]. Furthermore, proteomic studies revealed that activation of PDGF signaling is associated with Proneural phenotype [40]. PDGFRα is selectively expressed in OPCs in the normal brain [60]–[64], and the ability of PDGF signaling to stimulate the proliferation and migration of OPCs is well established [61], [64]–[69]. Similarly, several experimental models have shown that PDGF stimulation can induce the formation of gliomas [11], [12], [14], [17], [22], [42]. In particular, one study using the RCAS/tv-a system has shown that PDGF is sufficient to induce neonatal OPCs to form gliomas that resemble low-grade oligodendrogliomas [21]. In our model the tumors show the histological features of GBM. Furthermore, we show that these tumors have a Proneural phenotype and are also highly enriched in expressing OPC genes. While it is possible that PDGF signaling plays a role in inducing the expression of Proneural genes, our results suggest that PDGF responsiveness is an inherent feature of the cells that give rise to Proneural GBM. Thus, the role of PDGF signaling in OPCs and Proneural GBM provides a compelling example of how cancer cells can highjack specific signaling pathways that regulate the development of those cells from which they arise.

In addition to PDGFRα amplification, other genetic alterations, including p53 and IDH1 mutations, are strongly associated with Proneural GBM [10]. We propose that OPCs are particularly sensitive to the transforming effects of these genetic alterations. In this study, we demonstrated that PDGF stimulation and tumor suppressor Pten and p53 deletion cooperate to induce PDGFRα expressing adult glial progenitors to form GBM-like brain tumors. Using activated EGFR combined with p53 deletions, Persson et al. provided evidence that OPCs can be induced to form tumors resembling human oligodendroglioma [27]. The differences in tumor types seen in the previous studies and our study could be due to the nature of the genetic alterations introduced and/or the age of the animal when these alterations are delivered. Interestingly, it was shown that, in addition to a subset of GBM [10], oligodendrogliomas are also enriched in the Proneural signature [70]. Together, these results are consistent with the close relationship between OPC and the Proneural phenotype.

A recent paper showed that genetically deleting combinations of tumor suppressors (Pten, Rb1 and p53) in the adult brain with an inducible Cre system (GFAP-CreER) will give rise to high grade astrocytomas [71]. These tumors were widely distributed in the brain and showed a spectrum of phenotypes that resembled the different subtypes of high grade gliomas as described [7], [71]. They did not find a significant correlation between the types of genetic alterations used to initiate tumors and the subtypes of tumors that formed. However, they found an intriguing correlation with tumor location, suggesting that tumor subtype may be determined by microenvironment or by the type of cells that gives rise to the tumor.

### Heterogeneity within Proneural GBM is correlated with genetic alterations and clinical outcome

While Proneural GBM cluster into a common group based on the expression of 840 classifier genes, evidence also suggests that there is significant heterogeneity within Proneural GBM and that this heterogeneity may have clinical and prognostic relevance. Previously, using a list containing 35 genes, high grade glioma (HGG), including GBM, were classified into three groups: Proneural, Proliferative and Mesenchymal [7]. In this classification system, the Proliferative and Mesenchymal tumors were associated with poor prognosis, as defined by shorter patient survival, while Proneural tumors were associated with better prognosis. When the Phillips et al. classification system is applied to 59 TCGA Proneural GBM identified by Verhaak et al. classification system, 40 of 59 TCGA Proneural patients remained as Proneural, while the other 19 patients were classified as Proliferative (18) or Mesenchymal (1) [7], [10] (Figure 5B). Interestingly, when we mapped the results of both the Phillips and Verhaak classifications onto our GSEA ranking, the distribution of the 40 Proneural GBM identified by both classifiers was significantly skewed towards the end that more closely resembled Pten^f/f^ tumors in mice (Figure 5B).

Our two mouse models (Pten^f/f^ vs. Pten^f/f^; p53^f/f^) provide us a unique tool to understand heterogeneity within Proneural subtype of GBM. We generated a list of genes that are differentially expressed between these two mouse models. The list is highly enriched in genes involved in cancer, cell death and growth, and many of the genes are known to be functionally related to p53. Using this gene list discovered in mice, we separated human Proneural GBM into groups that more closely resembled one or the other mouse model. Strikingly, there was significant difference in survival between these groups, with those tumors that more closely resembled Pten^f/f^ mouse tumors having a greater than 2 folds longer median survival than those that resembled Pten^f/f^; p53^f/f^ mouse tumors. Therefore, Proneural GBM can be separated into prognostically distinct subgroups based on their similarity to our mouse models.

Analysis of the TCGA dataset suggests that mutation status of p53 alone does not predict survival in Proneural GBM. However, the p53 pathway can be compromised by alterations other than mutations in p53 itself, such as MDM2 amplification [72]. Although our DGE list was identified by comparing tumors with and without p53 deletion as an initiating genetic lesion, this gene list likely captures the global changes in expression signatures that result from overall alterations in p53 signaling.

Recently, a report identified a group of TCGA GBM patients with a glioma-CpG island methylator phenotype (G-CIMP) [73]. The majority of these patients belongs to the Proneural subtype and is associated with longer survival [73], [74]. Interestingly, these G-CIMP+ patients are evenly distributed on our GSEA ranking (Table S1). Furthermore, removing these patients from the data set caused a similar reduction in median survival from both groups, but with limited overall impact on the difference in survival (resembled Pten^f/f^ vs. Pten^f/f^; p53^f/f^: median survival 448d vs. 238d, p = 0.0315). This suggests that the heterogeneity within Proneural GBM that has been revealed by our approach is independent of their G-CIMP status. As is the case with many prognostic factors identified through analysis of human brain tumors, G-CIMP status shows a strong correlation with patient age. In contrast, the gene list identified in our study separates Proneural GBM patients into prognostically distinct groups that are independent of age (Table S1). This is likely due to the fact that our approach allowed us to control for animal age at the time of tumor induction. Together, these results indicate that p53 signaling alterations play a central role in the malignant transformation of OPCs as well as in determining aggressiveness of Proneural GBM.

### Conclusions

Our study reveals a close relationship between OPCs and Proneural GBM. These findings suggest that understanding the mechanisms that regulate OPC proliferation, differentiation and survival may lead to new therapeutic targets tailored towards Proneural GBM. In the process of this study, we also generated remarkably robust and consistent GBM models in mice, with built-in luciferase reporters that allow real time imaging of tumor growth. These models can be used to test the efficacy of new therapies directed towards Proneural GBM.

## Methods

### Ethics statement

All experimental procedures involving mice were approved by the Institutional Animal Care and Use Committee (IACUC) of Columbia University and performed in accordance with institutional policies.

### Retrovirus construction and intracerebral stereotactic injections

PDGF-IRES-Cre was generated by cloning human PDGF-B [42] and Cre [75] into pQXIX vector (Clontech). Cre-GFP was described [76]. VSVG pseudotyped retrovirus was generated as described in Methods S1. Cre-GFP retrovirus was titered to 10^7^/ml in 293 cells. PIC retrovirus was titered to 10^6^/ml using Mouse Embryonic Fibroblasts (MEFs) prepared from stop-floxed YFP mice [77]. Brain surgery procedures in mice were as described in Methods S1. For WM targeting, we used the coordinates of 2.1 mm lateral, 2.2 mm rostral and 1.8 mm deep, (2.1 mm+2.2 mm+1.8 mm) with bregma as the reference point. For targeting dorsal lateral corner of SVZ, we used the coordinates of 1 mm+1 mm+2.1 mm, with bregma as the reference. Using a stereotaxis platform, 0.4 µl retrovirus was injected into the brain with a Hamilton syringe (flow rate 0.1 µl/min). For serial transplantation experiments, 2×10^4^ primary tumor cells were re-suspended in 2 µl of Opti-MEM (Invitrogen) and injected into brain with flow rate at 0.2 µl/min. The coordinates for cell injection were 2 mm+2 mm+2 mm, with bregma as the reference.

### Transgenic mice

Mice harboring floxed tumor suppressors and stop-floxed reporters were generated by breeding the following strains: floxed Pten mice [78], floxed p53 mice [79], stop-floxed YFP mice [77] and stop-floxed luciferase mice (Thomas Ludwig, unpublished). The resulting mouse lines therefore are on mixed genetic backgrounds. NOD/SCID mice were purchased from Jackson Labs. All injections were done in mice between six and eight weeks of age.

### Bioluminescence imaging

100 µl of 30 mg/ml luciferin (Caliper Life Sciences) was injected intraperitoneally into each mouse. The IVIS Spectrum (Caliper Life Sciences) was used to capture bioluminescence images. All mice were imaged between 10 to 15 minutes after substrate injection (with 1 minute exposures). Mice with active tumor growth were imaged at least once per week, while control mice were imaged monthly. All control mice were last imaged at the conclusion of survival study.

### Brain sectioning - histological and immunohistochemical analysis

Mice were transcardially perfused with 15 ml ice cold PBS, followed by 15 ml cold 4% paraformaldehyde (PFA). Brains were removed and fixed overnight at 4 degrees in 4% PFA. Brains were paraffin embedded and microtome sectioned (5 µm thick) and then processed for histological analysis with hematoxylin and eosin stains, or, cryoprotected in 30% sucrose, snap frozen in OCT, cryosectioned (10 µm or 50 µm thick) and then processed for immunofluorescence analysis. Serial sections were collected and analyzed from at least 5 mice in each experimental group.

### Antibodies and confocal imaging

Primary antibodies used were as follows: Mouse-GFAP (Chemicon), Rab-GFAP (Dacocytomation), Rab-Olig2 (Chemicon), Guinea pig-Olig2 (Gift of Dr. Tom Jessell), Rab-PDGFRα (Cell Signaling), Rab-Pten (Cell Signaling), Rab-GFP (Invitrogen), Chicken-GFP (Invitrogen), Rab-ki67 (Vector Lab), Rab-ki67 (Abcam), Guinea pig-NG2 (Gift of Dr. Bill Stallcup), Rab-NG2 (Chemicon), Goat-Doublecortin (Santa Cruz), Rab-Sox2 (Chemicon), Mouse-NeuN (Chemicon), Rab-Tcf4 (Millipore), Mouse-Nkx2.2 (DSHB), Rab-Sox10 (Sigma), Mouse-Nestin (Chemicon), Hoechst 33342 (Invitrogen). Secondary antibodies were purchased from Invitrogen, conjugated with Alexa 488, 568 or 647. Z series confocal images were performed using a Zeiss LSM 510 Meta under 40× oil objective with 1 µm incremental steps. Z series was then stacked together to generate the projected view. All confocal pictures shown were projected views. These images were imported and processed in ImageJ and Adobe Photoshop.

### Primary tumor preparation and staining

Primary tumor preparation was generated as described in Methods S1 [17]. The tumor preparation was plated overnight on poly-lysine coated dishes. For serial transplantation studies cells were injected into NOD/SCID mice (as described above). For immunocytochemical staining, 3×10^4^ cells per well were plated on 8 well culture chamber slides overnight. Cells were then fixed and stained the next day as described [17].

### Gene Expression

End stage mouse tumors were dissected and snap frozen, and then shipped on dry ice to Bionomics facility in Rutgers University for expression array analysis. The platform used was Affymetrix® GeneChip® Mouse Genome 430A 2.0 Array (Affymetrix®, Santa Clara, CA). The microarray labeling, hybridization and quality controls were performed by following Affymetrix® protocol. Raw data was then normalized and summarized by robust multichip average (RMA) [80]. The dataset for different neural lineages in the mouse brains was downloaded from NCBI GEO with the access number GSE9566 [43]. The complete human GBM datasets were downloaded from TCGA website (cancergenome.nih.gov), NCBI GEO with the access number GSE4271, Rembrandt website (https://caintegrator.nci.nih.gov/rembrandt/) and Broad Institute (http://www.broadinstitute.org/cgi-bin/cancer/publications/pub_paper.cgi? mode=view&paper_id=162&p=t) [7], [10], [44], [45].

### Mouse and human expression comparison

Microarray data from mouse tumors and TCGA tumors were summarized and normalized separately. The probes were summarized to gene level by selecting the probe showing the highest variability measure as inter-quartile range (IQR). A unified dataset was generated including 9934 genes. To identify the human to mouse orthologs, we utilized the gene level sequence based mapping method, which is based on the reciprocal best match as available from NCBI HomoloGene (build64) [81], [82]. The data was centered around zero by performing a z-score linear transformation of the expression values. Given the highly homogenous nature of the mouse samples, we used an across genes standardization, which provided an optimal estimate of the standard deviation. This transformation, although still affected by many variables, makes it possible to directly compare the expression values to the classifier centroids and to run a rank correlation based clustering analysis. This dataset was then hierarchically clustered in an unsupervised manner using Pearson Correlation and average linkage without leaf order optimization, limited to the ortholog classifier genes (723 out of 840), and heatmap was generated to allow visual representation of gene expression and samples segregation as published [83], [84]. We used TMEV version 4.6 (www.tm4.org).

### Classification

We applied two approaches to classify the mouse samples, both of which used the mouse orthologs mapped from the original human classifier genes [10]. In the first approach we modified the centroid classifier as published [10]. We re-computed the centroids using 723, instead of 840, classifier genes. We used the nearest centroid-based classification algorithm ClaNC [85] to retest the human validation set and to predict the mouse samples. The mis-classification rate in the original human validation set was below 10% compared to what was previously published [10]. In the second approach, we again used the human training set, and then averaged the expression values of each gene across samples belonging to the same GBM subtype. We computed the Pearson correlation coefficients between the given query mouse sample and each GBM subtype and assigned it to the class with which it showed the highest correlation coefficient. These results also allow us to validate the classification results obtained using the modified ClaNC classifier [10], [85]. We performed this analysis for both the mouse samples and the human validation set (Table S1), and the error rate was again below 10% in the human set. With both methods, the classification of all samples matched.

### Comparing mouse tumors and the brain lineage datasets

We used expression data from the murine brain transcriptome database as published [43], which includes Gene sets that are specifically enriched in the following cell types: astrocytes, oligodendrocytes, neurons, OPCs, and cultured astroglial cells (presented in Table S4, S5, S6, S17 and S21, respectively of Cahoy et al, 2008). Each of these 5 gene sets contains 80 genes that are enriched greater than 1.5- fold in the respective cell type. The OPC list represented genes enriched greater than 1.5-fold in OPCs compared to myelinating Oligodendrocytes. The cultured astrocytes list includes genes enriched greater than 1.5-fold in cultured astroglia compared to *in vivo* astrocytes. 369 of 400 genes were mapped to the Affymetrix® GeneChip® Mouse Genome 430A 2.0 Array. We normalized and summarized together our mouse array raw data and the dataset GSE9566 with RMA. We computed an unsupervised hierarchical clustering of the combined dataset using the gene list (369) of different neural lineages. All gene lists of this study are provided in Table S1.

### DGE and Ingenuity

To identify the statistically significant differentially expressed genes between the two mouse models we used the limma package [86]–[88] within R/Bioconductor framework [89]–[93], due to its high sensitivity and increased control for false positive rate compared with other variance modeling strategies [94]–[97]. For this specific analysis we normalized the mouse samples using gcRMA due to its optimal combination between precision and accuracy [98], [99]. For the Ingenuity Pathway Analysis® (www.ingenuity.com), we used the genes lists from the differential expression analysis, at False Discovery Rate 0.1 cutoff, to identified biological function and diseases that were enriched. The corresponding p value was calculated with Fisher's exact test (Ingenuity reference user guide). The network connections were identified using Ingenuity Pathways Knowledge Base.

### GSEA

GSEA [100], [101] of the murine neural lineage genes [43] in the integrated mouse and human GBM data set was conducted as described [10] and implemented in a matlab script [102]. Expression values were replaced by ranks and the reported Enrichment Score (ES) was computed as sum of the maximum deviations above and below zero in the random walk. We set the exponent to the rank as weight to 1/5 according to the number of gene classes. GSEA was also used to rank order the tumors on the basis of the DGE lists generated from comparing two mouse models. The ES from each gene list was z-scored and the difference was used to rank-order the samples. The single sample GSEA was conducted using the GSEA implementation on the Broad Institute (www.broadinstitute.org/gsea/download) with DGE gene lists.

### Survival Analysis

Kaplan-Meier survival analysis and the Mantel-Cox log-rank test were performed using Prism 4 (Graphpad software).

## Supporting Information

Figure S1 Serially transplanted tumors retained capacity to form GBM.
(DOC)

Figure S2 Immunophenotypes of tumor cells are consistent with OPC identity.
(DOC)

Figure S3 Most cells infected by PDGF retrovirus express markers of OPCs by 17 dpi.
(DOC)

Figure S4 Injecting PDGF retrovirus into SVZ infects progenitor cells that gave rise to olfactory neurons.
(DOC)

Figure S5 PDGF driven mouse tumors resemble human Proneural GBM and express signatures of OPCs.
(DOC)

Table S1 (XLS)

Methods S1 (DOC)
