# Supplementary material for: Glioblastoma Models Reveal the Connection between Adult Glial Progenitors and the Proneural Phenotype
Source: PLoS One. 2011 May 23;6(5):e20041. doi: 10.1371/journal.pone.0020041 (PMC3100315; doi:10.1371/journal.pone.0020041)
Supplement: Figure S1 — Serially transplanted tumors retained capacity to form GBM. (DOC) [file pone.0020041.s001.doc]

Figure S1


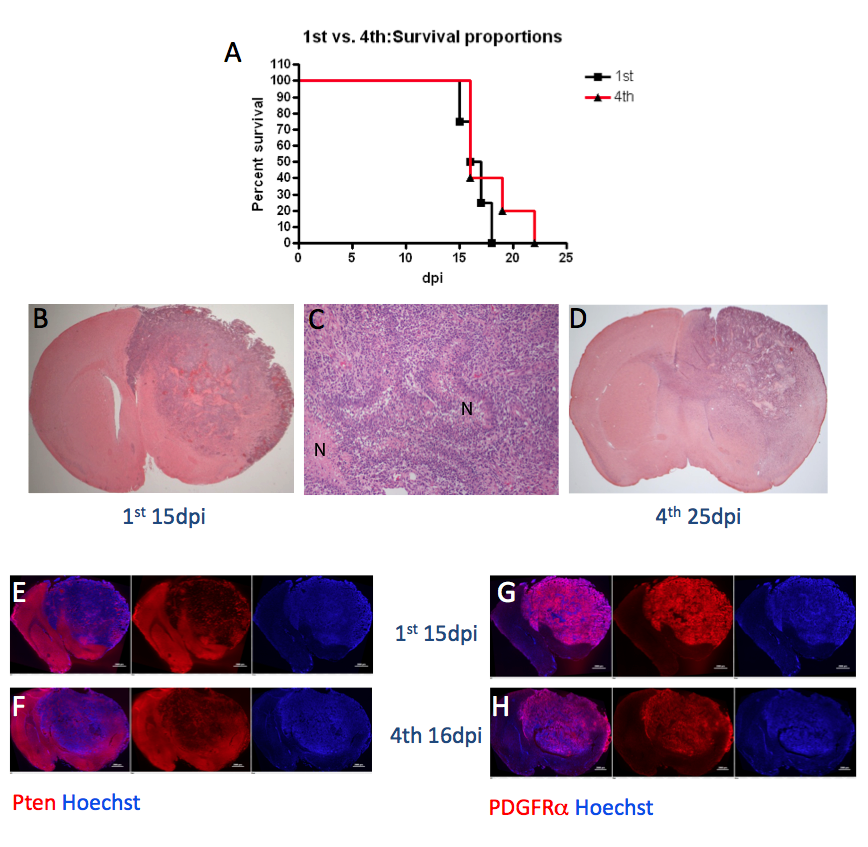


Figure S1. Serially transplanted tumors retained capacity to form GBM. Cells isolated from Pten^f/f^; p53^f/f^ tumors were serially transplanted into the brains of naïve adult NOD/SCID mice for up to 4 generations. (A) Kaplan-Meier survival curve of serial transplanted tumors: 1^st^ vs. 4^th^ generation p=0.2752. (B) Histology of 1^st^ generation serial transplanted tumor resembles GBM. (C) Magnified view of transplanted tumors show regions of necrosis and vascular proliferation. (D) Histology of 4^th^ generation transplanted tumor. (E) The 1^st^ generation tumors were mostly composed of Pten negative cells. (F) The 4^th^ generation tumors were mostly composed of Pten negative cells. (G) The 1^st^ generation tumors retained PDGFRα expression. (H) The 4^th^ generation tumors retained PDGFRα expression.
