# Supplementary material for: Glioblastoma Models Reveal the Connection between Adult Glial Progenitors and the Proneural Phenotype
Source: PLoS One. 2011 May 23;6(5):e20041. doi: 10.1371/journal.pone.0020041 (PMC3100315; doi:10.1371/journal.pone.0020041)
Supplement: Figure S2 — Immunophenotypes of tumor cells are consistent with OPC identity. (DOC) [file pone.0020041.s002.doc]

Figure S2


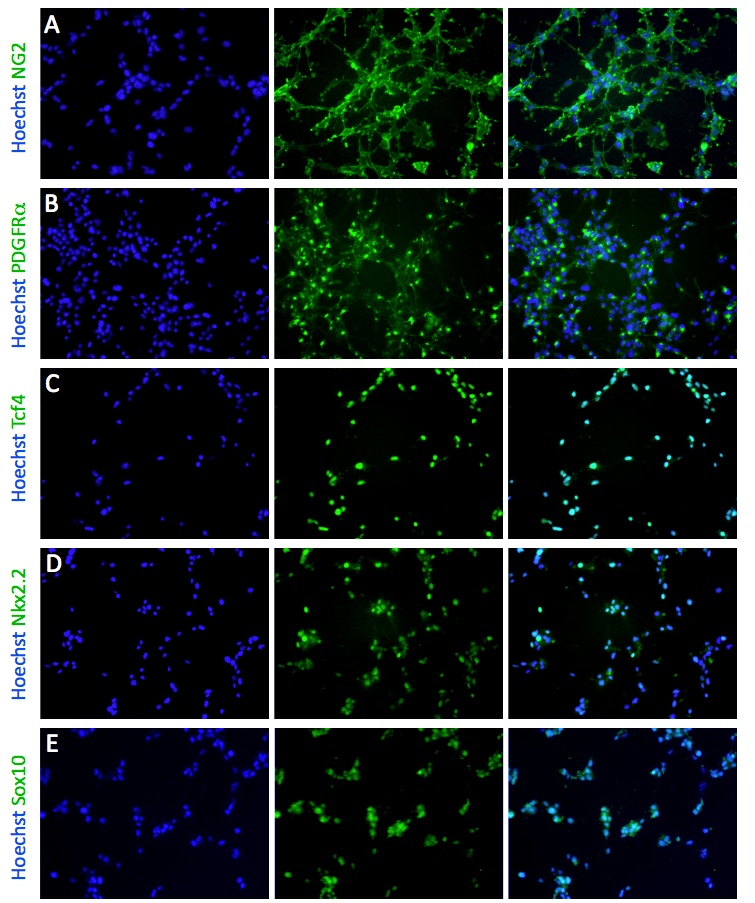


Figure S2. Immunophenotypes of tumor cells are consistent with OPC identity. Immunofluorescence analysis was performed on cells isolated from Pten^f/f^; p53^f/f^ tumors. (A) Tumor cells express NG2. (B) Tumor cells express PDGFRα. (C) Tumor cells express Tcf4. (D) Tumor cells express Nkx2.2. (E) Tumor cells express Sox10.
