# Supplementary material for: Glioblastoma Models Reveal the Connection between Adult Glial Progenitors and the Proneural Phenotype
Source: PLoS One. 2011 May 23;6(5):e20041. doi: 10.1371/journal.pone.0020041 (PMC3100315; doi:10.1371/journal.pone.0020041)
Supplement: Figure S3 — Most cells infected by PDGF retrovirus express markers of OPCs by 17 dpi. (DOC) [file pone.0020041.s003.doc]

Figure S3


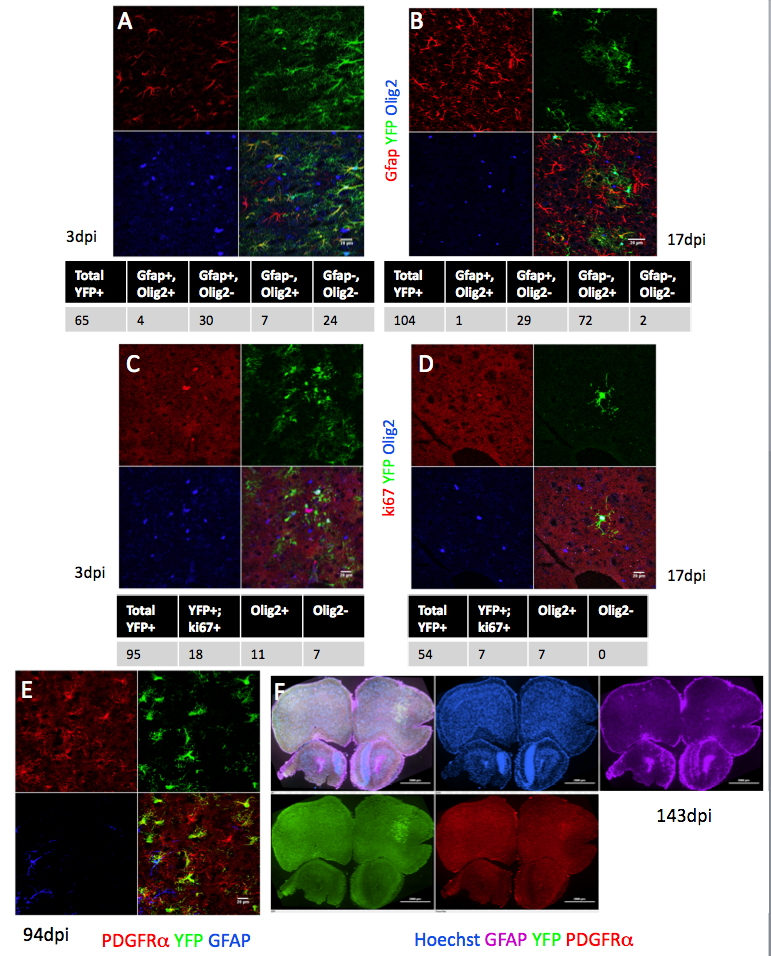


Figure S3. Most cells infected by PDGF retrovirus express markers of OPCs by 17dpi. PIC retrovirus was injected into the white matter of stop-floxed YFP mice with wild type Pten and p53. (A) Gfap and Olig2 profile at 3dpi: a fraction of YFP+ cells express Olig2. (B) Gfap and Olig2 profile at 17dpi: majority of YFP+ cells (~70%) express Olig2. (C) ki67 and Olig2 profile in YFP mice at 3dpi: more than 60% of YFP and ki67 double positive cells express Olig2. (D) ki67 and Olig2 profile in YFP mice at 17dpi: all YFP and ki67 double positive cells express Olig2. (E) Gfap and PDGFRα profile at 94dpi: most YFP+ cells express PDGFRα. No YFP+ cells express Gfap. (F) Montage view of Gfap and PDGFRα profile at 143dpi: YFP cells remain clustered closely. Most YFP cells express PDGFRα. No YFP+ cells express Gfap. There are no YFP+ cells in RMS or olfactory bulb.
