# Supplementary material for: Glioblastoma Models Reveal the Connection between Adult Glial Progenitors and the Proneural Phenotype
Source: PLoS One. 2011 May 23;6(5):e20041. doi: 10.1371/journal.pone.0020041 (PMC3100315; doi:10.1371/journal.pone.0020041)
Supplement: Figure S4 — Injecting PDGF retrovirus into SVZ infects progenitor cells that gave rise to olfactory neurons. (DOC) [file pone.0020041.s004.doc]

FigureS4
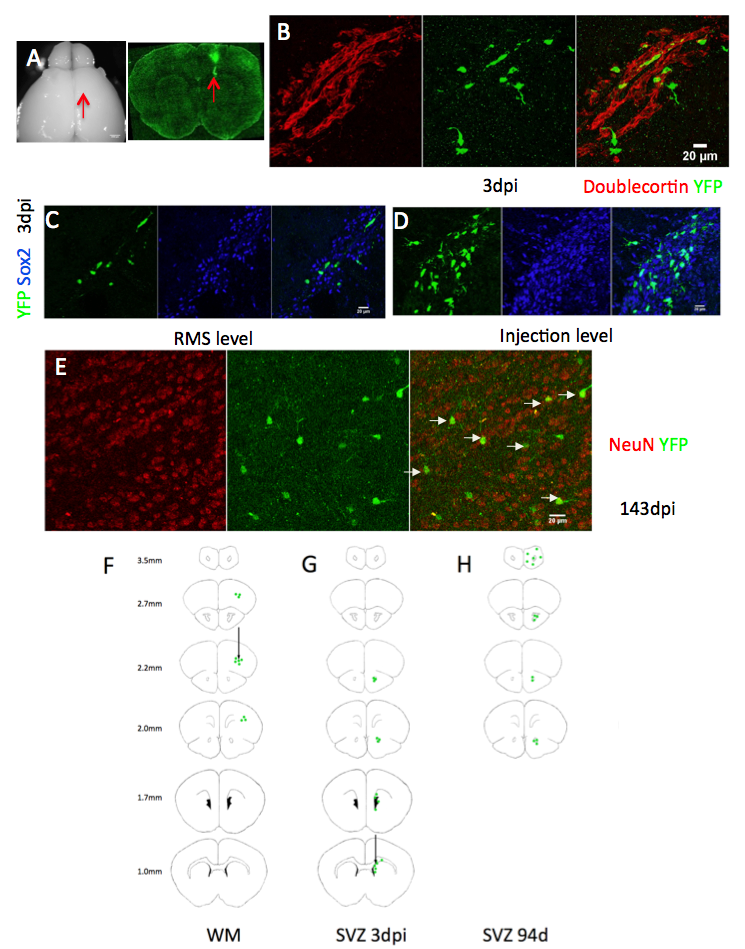


Figure S4. Injecting PDGF retrovirus into SVZ infects progenitor cells that gave rise to olfactory neurons. PIC retrovirus was injected into the dorsal lateral SVZ of stop-floxed YFP mice with wild type Pten and p53. (A) SVZ targeting validation using GFP virus: left panel shows the dorsal view of the brain, right panel show a coronal section at the level of the injection site. The red arrows mark the injection site (left panel) and needle track (right panel). (B) Injecting PDGF retrovirus into SVZ labeled cells in RMS at 3dpi: some, but not all, YFP+ cells are Doublecortin (red) positive. (C) Injecting PDGF retrovirus into SVZ labeled many Sox2 positive cells in RMS at 3dpi. (D) Injecting PDGF retrovirus into SVZ labeled many Sox2 positive cells around SVZ at 3dpi. (E) PDGF retrovirus infected cells gave rise to olfactory neurons at 143dpi: some YFP+ cells in the olfactory bulb express NeuN (red), a neuronal marker, as labeled by white arrow. (F) Schematic view of cell distributions in WM targeting: no retrovirus infected cells were observed in SVZ, RMS or OB at any time points. (G) Schematic view of cell distributions in SVZ targeting at 3dpi: retrovirus infected cells started migrating into RMS. (H) Schematic view of cell distributions in SVZ targeting at 94dpi: retrovirus infected cells were observed in OB. Green dots indicate distribution of YFP+ cells (F-G). Black arrows indicate the location of the injection site (F and G).
