# Supplementary material for: Glioblastoma Models Reveal the Connection between Adult Glial Progenitors and the Proneural Phenotype
Source: PLoS One. 2011 May 23;6(5):e20041. doi: 10.1371/journal.pone.0020041 (PMC3100315; doi:10.1371/journal.pone.0020041)
Supplement: Figure S5 — PDGF driven mouse tumors resemble human Proneural GBM and express signatures of OPCs. (DOC) [file pone.0020041.s005.doc]

FigureS5


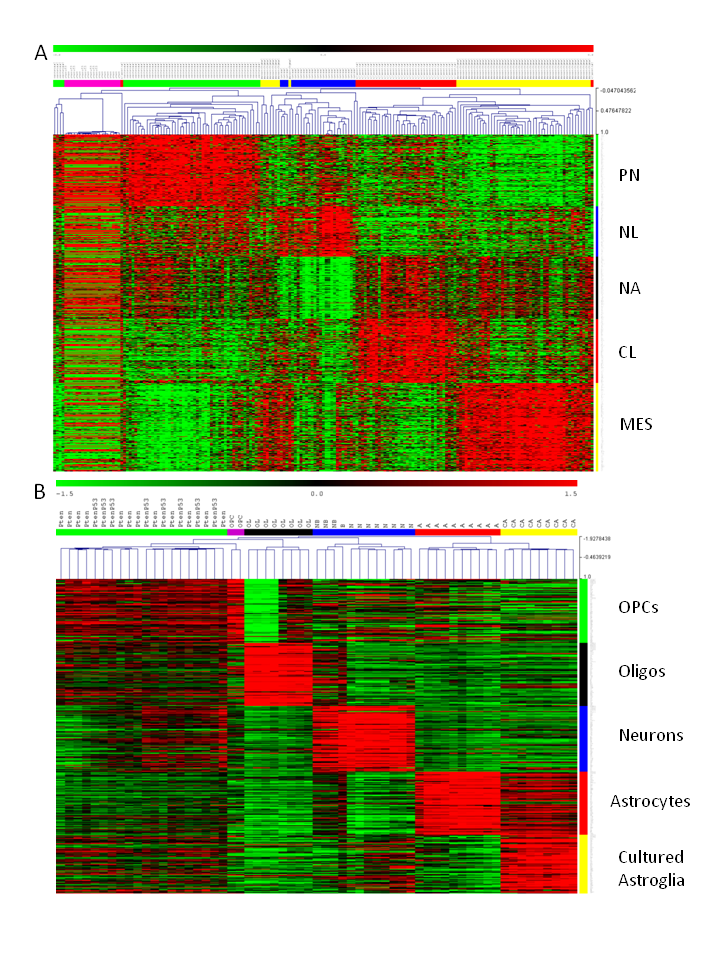


Figure S5. PDGF driven mouse tumors resemble human Proneural GBM and express signatures of OPCs

A: Unsupervised hierarchical clustering indicates mouse tumors cluster with Proneural GBM. Sample wise (horizontal): mouse tumors are labeled as purple, Proneural GBM as green, Neural GBM as blue, Classical GBM as red, Mesenchymal GBM as yellow. Genelist wise (vertical): Proneural genes (PN) are labeled as green, Neural genes (NL and NA) NL as blue and NA as black, Classical genes (CL) as red and Mesenchymal genes (MES) as yellow. The heatmap shows the expression of the 723 classifier genes across all tumor samples. Red corresponds to high expression and green corresponds to low expression.

B: Mouse tumors clustered with murine OPCs. Sample wise (horizontal): tumors are labeled as green, OPC as purple, oligodendrocytes (OL) as black, normal brain (NB), forebrain (B) and neurons (N) as blue, astrocytes (A) as red and cultured astroglia (CA) as yellow. Genelist wise (vertical): OPC genes (OPCs) are labeled as green, mature oligodendrocyte genes (Oligos) as black, neuron genes (Neurons) as blue, astrocyte genes (Astrocytes) as red and cultured astroglia genes (Cultured Astroglia) as yellow. The heatmap shows the expression of the 369 cell-type specific genes across all tumor samples. Red corresponds to high expression and green corresponds to low expression.
