# Supplementary material for: Glioblastoma Models Reveal the Connection between Adult Glial Progenitors and the Proneural Phenotype
Source: PLoS One. 2011 May 23;6(5):e20041. doi: 10.1371/journal.pone.0020041 (PMC3100315; doi:10.1371/journal.pone.0020041)
Supplement: Methods S1 — (DOC) [file pone.0020041.s007.doc]

Supporting Methods

Retrovirus production

293GP cells were seeded to ~70% confluence in complete medium (DMEM+10%FBS). Plasmid expressing VSVG and viral plasmid PIC were mixed with CaCl_2_. 2XHBS (Hepes Buffered Saline) was added to the mix, which was overlaid onto the cells. After overnight incubation, fresh complete medium was changed and collected 48 hours later. After filtration, the medium was centrifuged at 35,000 rpm for 1hr at 4^0^C in Ti45 rotor. The pellet was re-suspended in PBS, and viral aliquots were frozen and stored at –80^0^C until use. MEFs from stop-floxed YFP mice were used to titer cre-expressing retroviruses. Viral titers were determined by incubating cells with serial dilutions of retrovirus in 10-fold steps. YFP expressing cells were counted 48 hrs later. The viral titer was determined by the lowest dilution that gave rise to YFP positive cells.

Intracerebral stereotaxic injections

The mice were anesthetized with an intraperitoneal injection of Ketamine/Xylazine (100mg/kg and 10mg/kg respectively). After sufficient anesthesia was induced, the mice were placed in a stereotaxic frame. Approximately a 1cm incision was made in the midline of the scalp to expose bregma and sterotaxic coordinates were determined. A burr hole was drilled through the skull and a Hamilton syringe containing the retrovirus was then inserted into the brain. A volume of 0.4µl was injected at 0.1µL/min. At the end of the injection, the needle was slowly retracted. The skin was closed with 2 stitches of vicryl sutures. The animal was then removed from the stereotaxic frame. The entire length of the procedure was approximately 20 minutes. The suture was removed in 7 to 10 days. Following surgery the mice were placed on a warming pad to recover. In the immediate post-op period, the animal was continuously monitored until fully awake. Once the animal completely recovered, it was returned to the mouse facility. During the recovery period, animals were re-examined at 12 and 24 hours post-op. After the first 24 hours post-op, the observation schedule became once daily with weight, appearance, and behavior being monitored.

Survival studies

The effects of brain tumors on mice primarily involved the neurological deficits produced by an enlarging intracranial mass. In accordance with IACUC guidelines, animals were allowed to live until humane endpoints were reached. Signs of terminal tumor burden included the following; peri-orbital hemorrhages, papilledema, epistaxis (nose bleeds), seizures, decreased level of alertness, impaired motor function, and/or impaired ability to feed secondary to decreased motor function, paresis or coma. Prior to the onset of symptoms, the animals usually developed weight loss of at least 20 percent. Tumor growth was also monitored by serial luciferase imaging.

Primary tumor preparation

Ex-vivo gross total resection of the tumor was performed and the tissue was shredded and minced in a small amount of PBS. Enzymatic and mechanical dissociation followed a modified protocol [1]. The shredded tissue received a 30 minute digestive enzyme treatment in a 37 °C shaking bath. After the digestion, the dissociated cells were filtered through a 70µm mesh (BD Falcon). 5 ml of 10% heat-inactivated FBS was then added in order to inactivate the trypsin. Cells were centrifuged for 10 minutes at 450g and re-suspended in MEM containing 20m*M* HEPES and 30% sucrose. Cells were then centrifuged for 20 minutes at 770g and re-suspended in media. Tumor cells were plated onto 6-well tissue culture plates coated with poly-L-lysine at a concentration of 2 million cells per well. The cells were grown in culture media containing a 2:1 mixture of basal media and B104 conditioned media [2], further supplemented with 10 ng/ml PDGF-AA (Sigma) and 10 ng/ml FGFb (Gibco). Basal media contained N2 supplement (Gibco), 20 ng/ml T3 (Sigma), 0.5% FBS, and penicillin/streptomycin/amphotericin (Invitrogen) in DMEM (Gibco). B104 conditioned media was collected from confluent cultures of the B104 neuroblastoma cell line maintained in basal media for 48 hours.

Supporting References

1. Gensert JM, Goldman JE (2001) Heterogeneity of cycling glial progenitors in the adult mammalian cortex and white matter. Journal of Neurobiology 48: 75-86.

2. Canoll PD, Musacchio JM, Hardy R, Reynolds R, Marchionni MA, et al. (1996) GGF/neuregulin is a neuronal signal that promotes the proliferation and survival and inhibits the differentiation of oligodendrocyte progenitors. Neuron 17: 229-243.
